# An Interoperable Vaccine Record: A Roadmap to Realization

**DOI:** 10.3390/vaccines14030213

**Published:** 2026-02-26

**Authors:** Xia Jing, Arild Faxvaag, Christian G. Nøhr, David Robinson, Paul G. Biondich, Timothy D. Law, Hua Min, Adam Wright, Yang Gong, Dean F. Sittig

**Affiliations:** 1 Department of Public Health, College of Behavioral, Social and Health Sciences, Clemson University, Clemson, SC 29634, USA; 2 Department of Neuromedicine and Movement Science, School of Medicine, Norwegian University of Science and Technology, 7491 Trondheim, Norway; 3 Department of Sustainability and Planning, The Technical Faculty of IT and Design, Danish Center for Health Informatics, Aalborg University, 9000 Aalborg, Denmark; 4 Independent Researcher, Cumbria CA13 0RU, UK; 5 Department of Pediatrics, School of Medicine, Indiana University, Indianapolis, IN 46202, USA; 6 Global Health Informatics, Regenstrief Institute, Indianapolis, IN 46202, USA; 7 Ohio Musculoskeletal and Neurologic Institute, Ohio University, Athens, OH 45701, USA; 8 Department of Health Administration and Policy, College of Public Health, George Mason University, Fairfax, VA 22030, USA; 9 Department of Medicine, School of Medicine, Vanderbilt University, Nashville, TN 37232, USA; 10 Department of Clinical and Health Informatics, McWilliams School of Biomedical Informatics, The University of Texas Health Science Center at Houston, Houston, TX 77030, USA

**Keywords:** vaccination record, interoperability, standards, standard vocabularies, vaccination registry, challenges

## Abstract

**Objectives**: The objectives of this study were to educate the healthcare professional and the general public about interoperable vaccine records by elaborating on its definition, why we need one, what the challenges are, and what progress has been made in this direction. **Methods**: The vaccination practices and vaccine record-keeping in the Nordic countries, the UK, and the USA are used as examples to demonstrate the necessity of interoperable vaccine records. The authors’ expertise and experience in interoperability, medicine, and HealthIT, along with the literature, informed this paper’s content, structure, and organization. Real-world examples and scenarios illustrate the reality and significance of interoperable vaccine records. **Results**: This paper provides a brief description of vaccination records and their practices in the Nordic countries, the UK, and the USA, which can inform future best practices for vaccination record-keeping. This paper also proposes a conceptual roadmap for achieving an interoperable vaccine record, which is a critical component for maintaining the integrity of an individual’s health record longitudinally, an essential cornerstone for receiving safe and effective healthcare, improving patient outcomes, controlling healthcare costs, avoiding unnecessary revaccination (overvaccination), and enabling alignment with up-to-date vaccine recommendations. This paper examines the intersection of vaccinations, HealthIT, and vaccine record-keeping, and it provides a brief discussion of the social and political aspects of vaccination. **Conclusions**: Although achieving interoperable vaccine records is technically feasible and clinically important, their large-scale implementation is not a simple task amid the social and political challenges related to vaccine misinformation, acceptance, and hesitancy.

## 1. Background

### 1.1. Why Do We Need an Interoperable Vaccine Record?

Vaccination has been one of the most critical tools for keeping the general public safe, preventing many deadly infections, and also one of the most effective ways to improve life expectancy in the centuries since vaccination was discovered and implemented on a large scale [1]. In most of the world, vaccinations are recommended and administered for individuals from newborns to the elderly routinely, per each vaccine’s purpose, the individual circumstances (such as travel to high-risk areas or pregnancy), and the corresponding health department’s or professional societies’ recommendation, with explicit exceptions due to medical considerations and individual differences, primarily biological, psychological, or sociocultural factors [2]. However, the record of an individual’s vaccinations over a lifetime is typically not organized in one place, especially in the United States, even though proof of certain vaccinations is required for many public school enrollments.

In Nordic countries, however, national vaccination registries have been implemented for decades as a mandatory requirement for healthcare providers and professionals. For example, the Norwegian Immunization Registry SYSVAK started in 1995 [3], the Danish Vaccination Registry (DDV) [4,5] started in 2000, Iceland’s Vaccination Registry included e-vaccination records starting in 2002 [6], Finland’s National Vaccination Register (NVR) started in 2009 [7], and the Swedish national vaccination register (Svevac) started in 2012 [8]. The national vaccination registries in these Nordic countries serve as a central repository for each citizen’s vaccination history over their lifetime in each country, which provides a unique advantage compared to the vaccine records in the USA, primarily due to their different healthcare delivery systems and much broader cultural acceptance of vaccines. However, across Nordic countries’ borders or within European countries or a broader scope internationally, making their vaccination history portable is not a reality yet; therefore, an interoperable vaccine record is still a valid use case.

Meanwhile, in the UK, patients can view their health records, as captured by their general practitioners (GPs, an equivalent of primary care providers in the USA) [9]. Vaccination records are a part of such a health record. However, vaccinations can be given in a number of different contexts outside GP practices, for example, by health visitors (nurses who visit and monitor young children), midwives, pharmacists, or private travel clinics. Such vaccination records may not necessarily be incorporated into the GP’s health record, and it is unclear how portable these vaccination records generated outside GP practices are. Given that the National Health Service (NHS) covers all legal residents of the UK, the vaccination history of individual patients as captured by their GPs is very likely to be the largest portion. Meanwhile, there have been significant advancements in health information exchange (not necessarily interoperable) by the UK NHS [10]. Most recently, “medicine interoperability” is provided as an NHS service to make medication information interoperable across different information systems [11]. However, it is unclear whether vaccine records are interoperable yet in the UK.

**Figure 1:**
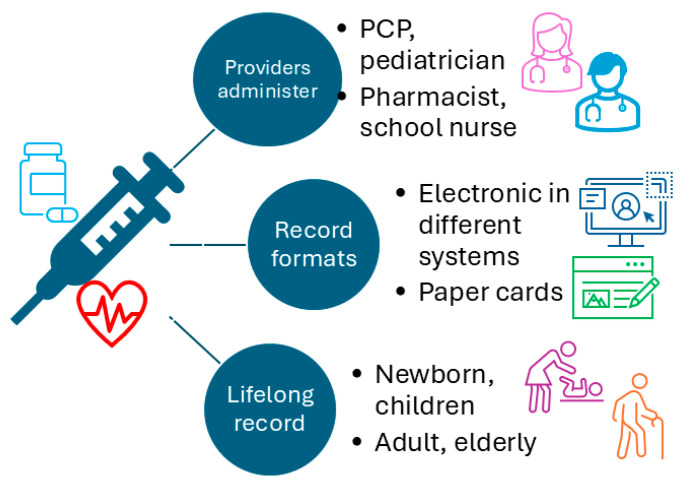
Current barriers to establishing a consolidated, longitudinal vaccination record (PCP, primary care provider).

The fact that many different types of healthcare providers in the USA (e.g., primary care providers, pediatricians, pharmacists, school nurses, and public health department nurses) can administer vaccines further fragments vaccination records. Moreover, many vaccine records are still on paper cards, which adds to the challenges of maintaining longitudinal vaccine records for every individual over their lifetime. In summary, the Nordic countries’ national vaccine registries are better organized than those in the USA; however, the portability of vaccine records across countries remains a challenge. The UK NHS has made significant progress in enabling patients to view their vaccination records via their GP; however, it is unclear whether vaccinations received outside the GP offices are also recorded in the GP’s records. Meanwhile, in the USA, vaccine records are maintained by multiple healthcare providers, and some are still in paper format, which adds an additional layer of complexity to achieving interoperability. Figure 1 illustrates the realities of vaccination records and the barriers to a consolidated, longitudinal vaccination record for everyone in the USA, the UK, the Nordic countries, and many other countries.

**Figure 2:**
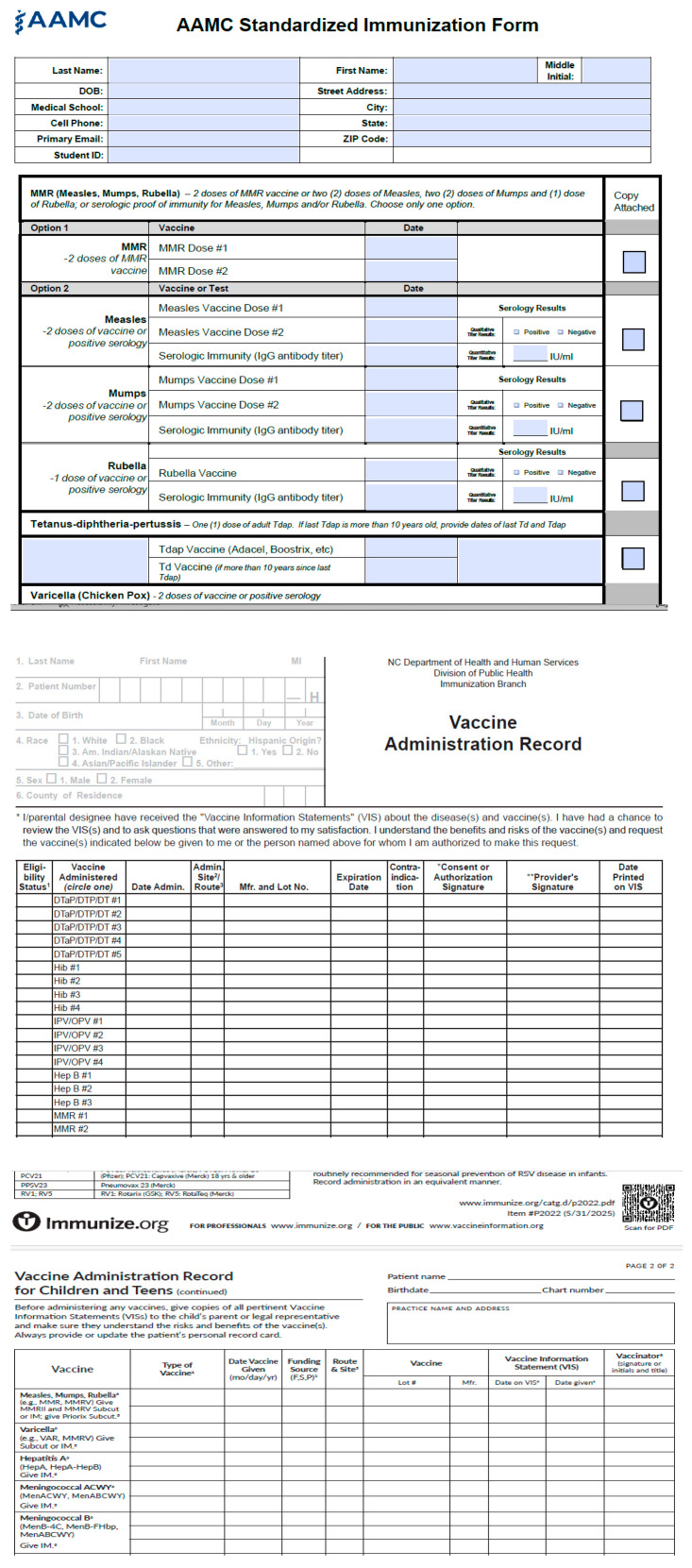
Vaccine record template examples from the American Association of Medical Colleges (**top**), the North Carolina Department of Health and Human Services (**middle**), and Immunize.org (**bottom**; all are publicly accessible resources).

Not only are there different types of healthcare providers who administer vaccines, but each type of provider is very likely to use a different template for vaccine records. We use several examples (Figure 2) to make the point. These examples are from the American Association of Medical Colleges (AAMC), the North Carolina Department of Health and Human Services, and Immunize.org (a non-profit organization). Each record has different fields for demographic information, and different names for the same vaccines (e.g., measles, mumps, and rubella, or MMR), and different options for the same vaccine; for example, the AAMC provides MMR and separate vaccines for measles, mumps, and rubella, which also include serology test results for each of the three vaccines. These examples have not yet considered the differences across countries or languages in vaccination records. All the differences demonstrate that achieving a consolidated, interoperable, and complete vaccination record is not a trivial or simple straightforward task.

### 1.2. What Is Interoperability, and Why Is It Important?

Interoperability is the capability of communicating and exchanging information among different information systems or applications and ***using*** the information after communicating it [12,13]. Although there are many mature information and communication technologies, and interoperability is common in many fields, such as banking, the airline industry, and retail, many of these are in the business world. However, in the healthcare field, using information after communication or exchange without additional manual effort is not yet a common practice, despite significant progress. For example, the UK NHS’s “medicines interoperability” is a significant progress in this direction [11]. This is primarily due to well-recognized challenges in healthcare and medicine, such as complicated workflows, the complex nature of clinical data, and ambiguous concepts, leading to challenging information modeling [12,13]. A recent national survey in the USA showed that only 18% of family physicians have ideal immunization-related interoperability, and the overall ideal interoperability is between 8% to 19% [14]. Interoperability can play a critical role in providing complete patient records for medical referrals, second-opinion consultations, hospital admissions and discharges, emergencies, relocations, and travel, all of which directly contribute to safer, timely, more effective, non-redundant clinical care, better outcomes, and reduced cost [15,16,17,18,19,20,21,22].

### 1.3. Significance of an Interoperable Vaccine Record

**Figure 3:**
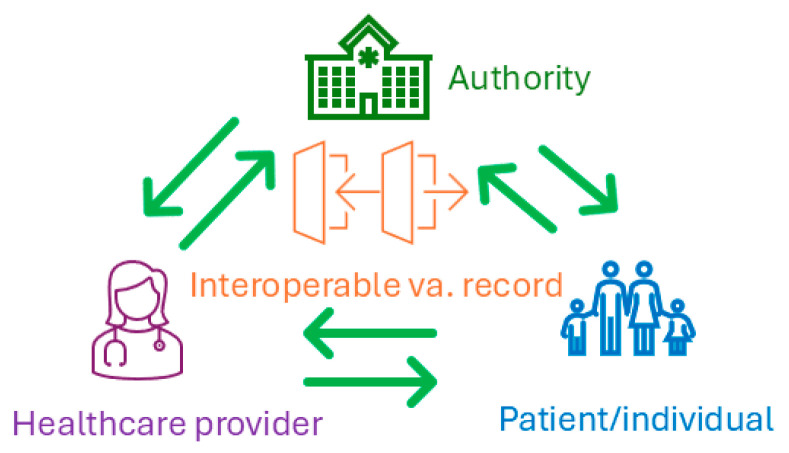
The primary stakeholders of interoperable vaccination records (authority can include health departments, schools, or trusted blockchain brokers; healthcare providers can include physicians, nurses, pharmacists; patient/individual can include caregivers; va., vaccination).

Within the vaccination record context, interoperability can not only help establish a longitudinal individual vaccination record more conveniently, but it can also enable the vaccination record to be easily moved among the three primary categories of stakeholders (Figure 3). Figure 3 shows the primary stakeholders of vaccination records, including individuals (such as parents for infants and young children, young adults, elderly, and sometimes adult children or a caregiver of the elderly), healthcare providers (such as primary care providers, pediatricians, pharmacists, public health department nurses or physicians, school nurses, geriatricians), and authorities (such as public schools, health departments, assisted living, long term care facilities, other institutional facilities, or ***trusted blockchain brokers*** [23]). Healthcare providers typically administer vaccines to individuals and keep their records within their own systems, with or without a copy to individuals; authorities request healthcare providers or individuals to provide vaccine records to meet administrative requirements, for example, attending public schools; individuals keep the paper cards with their administered vaccines and administration dates (sometimes including locations), and administers, which are very likely in a non-uniform format.

Here, we present two scenarios to illustrate the tangible benefits and practical significance of interoperable vaccination records for individuals and healthcare providers.

Scenario one is that an interoperable vaccination record can help establish a consolidated longitudinal vaccination record for each individual. For most individuals, from birth to the end of life, many vaccines will be administered. However, vaccination records were typically organized by parents before the individuals became adults, and by the individuals themselves and caregivers when the individuals became elderly and needed care. The nature of one’s growth trajectory and the different vaccines administered over one’s lifespan make the consolidated vaccination record challenging but necessary. In the Nordic countries, their national vaccination registries serve as a consolidated lifelong vaccine record for each citizen. In the UK, GP records capture most citizens’ vaccination records. However, the centralized solution is unlikely to become a reality in the USA any time soon. A complete record of all administered vaccines is particularly important for individuals with a compromised immune system or who are susceptible to vaccine or medication-related adverse events. Without an accurate vaccination record, both healthcare providers and patients face unnecessary risks of missed (i.e., under-vaccination) or duplicate vaccinations (i.e., over-vaccination), increased exposure to vaccine-preventable diseases, and compromised clinical and public health decision-making. It is unlikely that we can achieve a consolidated vaccination record with the current system, i.e., different record formats in paper and electronic versions across isolated systems owned by different healthcare providers. In addition, the realities of globalization, mobility, and travel create a common, more urgent need for a longitudinal vaccination record, which is critical for determining whether booster doses, revaccination, or additional vaccine administration are necessary. With an interoperable vaccination record, individuals (or their caregivers) can easily import or export the vaccination record across platforms within their own records, with their providers, and with the authority if needed (Figure 3). Here, we want to emphasize the ***bidirectional*** features of an interoperable vaccination record among the three primary stakeholders, which is a key difference and advancement compared to a pure electronic vaccine record.

Scenario two is for healthcare providers to submit vaccine records to the authorities to meet the administrative requirement more conveniently. Currently, particularly in the USA and many Western countries, it is common for healthcare providers to use electronic systems in routine healthcare delivery, including vaccine administration. However, such systems are not necessarily connected to those used by the authorities, such as health departments, school districts, or trusted blockchain brokers. Therefore, reporting vaccination records from healthcare providers to the authority is a common practice, an official requirement for collecting accurate population-level data, and a responsibility of healthcare providers. With an interoperable vaccination record, healthcare providers’ submissions and the authorities’ data collection can be streamlined, more convenient, and in real time. This benefit is not applicable to most healthcare providers in the Nordic countries, as they have already reached this point through their current national vaccination registries. However, in the UK, this functionality can still benefit the vaccines administered outside of GP practices. This functionality will greatly benefit the USA healthcare providers and authorities during their vaccination data submission, collection, and verification processes.

### 1.4. Contribution of This Paper to the Existing Literature

We propose the concept of the “interoperable vaccine record” for the first time as a natural extension of healthcare interoperability. This paper contextualizes the existing infrastructure within the interoperable vaccine record framework and articulates the necessity of an interoperable vaccine record across countries, drawing on examples from the Nordic countries, the UK, and the USA, on progress toward interoperability, and on the challenges that remain. This paper can inform healthcare professionals, technical personnel, and the general public about what an interoperable vaccine record is, how it differs from an electronic vaccine record, and the necessity of such a record. It can also guide future efforts to achieve interoperable vaccine records. However, this manuscript provides a conceptual-level roadmap rather than a day-by-day implementation plan with actionable steps.

### 1.5. Methods Used

We used the Nordic countries, the UK, and the USA as examples to compare the vaccination practices and vaccine record-keeping. A convenient sample was obtained among the coauthors, aiming to demonstrate the realities in vaccination and the necessity of interoperable vaccine records. All the authors’ decades of experience and expertise in interoperability, medicine, and HealthIT, as well as the existing literature, have informed this paper’s content, structure, and organization. In this manuscript, healthcare providers refer to healthcare professionals who can administer vaccines and maintain their records; individuals are used interchangeably with patients or citizens, i.e., recipients of vaccines, who may need interoperable vaccine records for travel, relocation, or school enrollment; and authority refers to government at different levels (school, county, state, national, or international) or the trusted brokers, such as those enabled by blockchain technology; authority typically establishes the rules related to vaccine requirements and enforces their implementation.

## 2. Existing Supportive Infrastructure

### 2.1. CDC and Other Professional Societies’ Recommendations on Vaccination

The USA Centers for Disease Control and Prevention (CDC) is a premier worldwide public health institute and a USA federal government agency that serves Americans at the individual, family, and community levels through accurate data, health guidance, and preventive measures, including vaccination schedules. Many CDC recommendations are adopted and implemented by USA groups (clinicians, policy makers) and beyond [2]. Therefore, the impact of the CDC is beyond the USA national border. For vaccination in particular, the two recommended schedules are for kids (i.e., younger than 7 and 7 to 18 years old) and adults (i.e., 19 years and older), the two target populations; the schedules are prepared in two formats, for healthcare professionals and parents, customized based on the users’ backgrounds. The recommendations are updated annually by consensus of an expert panel of pediatrician scientists. The CDC vaccination schedules can serve as a valuable source of knowledge for building computable technology artifacts that can be used within an EHR or other information systems to provide actionable recommendations in a clinical setting. However, the most common utilization of the recommendations is by the healthcare professionals’ direct comprehension, without computational transformation or processing. Meanwhile, in the USA, especially recently, more and more healthcare providers and insurance companies have adopted professional societies’ recommendations on vaccination schedules, such as those of the American Academy of Pediatrics and the American Academy of Family Physicians, to ensure healthcare providers can help patients and their families to make the most informed decisions on vaccinations.

### 2.2. State Immunization Registries in the United States

Immunization Information Systems (IIS) are population-level immunization record databases that record all the immunizations administered by participating healthcare providers in the USA [24]. The system is typically organized and maintained by individual states or jurisdictions [25], and the CDC maintains a registry of IIS, their URLs, and contacts. Some states have their own immunization registry, such as SIMON (Statewide Immunization Online Network) in South Carolina [26] and CHIRP (Children and Hoosier Immunization Registry Program) in Indiana [27]. The statewide immunization registry and the state IIS are typically interconnected or consolidated rather than operating in silos, a critical achievement that supports interoperability. IIS can serve as an official immunization record source; however, only participating providers’ records are included. Therefore, in theory, there are missing records in the official databases (IIS) by default. Despite potential missing records, IIS, when aggregated, can still serve as a population-level surveillance tool for vaccination, providing critical evidence for policymaking and operations to keep the general public safe and healthy.

The Immunization (IZ) Gateway [28] is another CDC resource and infrastructure that can provide some of the functionality of an interoperable vaccination record, i.e., the connection between the authority and the healthcare provider, and individuals can query their immunization records from the authority (Figure 3). However, the connections ***between the providers and the individuals,*** and ***from the individuals to the authorities,*** in Figure 3 (i.e., 50% of the functionalities) are not supported by the current IZ Gateway.

### 2.3. Electronic Patient Records Within the Healthcare System

Digital technology has been broadly adopted in healthcare; one demonstration is the increasing use of electronic health records in more than 96% of the hospitals (2021 data) [29] and over 95% of the physician offices (2024 data) [30] in the USA, a significant increase in office-based physician practices and non-academic health centers over the past decade or so [31]. The availability and accessibility of digital patient records not only provide critical technical infrastructure for interoperable vaccination records but also social and cultural preparation and literacy among patients, the general public, healthcare providers, and policymakers.

### 2.4. Standards and Vocabularies for Vaccination Records

Standards and controlled vocabularies are cornerstones for achieving semantic interoperability, including interoperable vaccination records. HL7 [32,33] is an international standards development organization and one of the largest and most diverse creators, maintainers, and owners of healthcare standards. Among all HL7 standards, the messaging standards and HL7 FHIR [33] (Fast Healthcare Interoperability Resources) are the most relevant to interoperable vaccination records. Meanwhile, CVX codes (for vaccines), SNOMED CT [34] (for diagnoses), and LOINC [35] (for lab test results) can all play a critical role in the consistent and unambiguous naming of vaccines, conditions, diagnoses, lab tests, and lab test results, all of which are necessary for a universal understanding of the semantics of vaccination records and, furthermore, for achieving interoperable vaccination records.

## 3. What Has Been Achieved in Computerizing Vaccination Records

### 3.1. Computable Vaccination Artifacts

Given vaccination’s effectiveness in keeping the general public safe and healthy, and the broad adoption and implementation of vaccination, vaccination schedules have long been a popular topic among researchers seeking to automate the vaccination scheduling process. Perry Miller and colleagues were pioneers in developing computer-based guidelines for childhood immunization [36,37,38,39,40,41,42,43]. They explored the representation, validation, testing, and maintenance of computer-based immunization guidelines in operating information systems, as well as the handling of incomplete patient histories to determine vaccination status. Other efforts have focused on translating immunization guidelines into scalable, computable decision-support tools, such as the immunization calculation engine (ICE) [44]. The CDC also has a mobile app to record and organize vaccines administered; however, manual data entry is required to populate the records, which can be a major barrier to broader adoption. Canada also released a national immunization app to leverage the high usage of smartphones among the general public [45]. Our group has explored interoperable clinical decision support system (CDSS) rules using childhood vaccination schedules [46,47,48,49,50]. The progress on reusable CDSS rules, a type of technical artifact, via leveraging ontologies, HL7 FHIR, and clinical quality language (CQL) presented its feasibility and established an important foundation for realizing interoperable vaccination records.

### 3.2. Other Work-Related Vaccination Records

Sorg and Khobzi examined a decade of usage data for the electronic vaccination record in Switzerland [51]. However, the results do not look encouraging. Although the authors identified regional factors, healthcare providers play a critical role in the success of such records. Other efforts include the creation of digital immunization records, incentivized by increased travel and global mobilization [52], a popular reason to promote electronic vaccination records. However, what we want to emphasize is beyond an electronic version of vaccination records; rather, interoperability is the core, which, if achieved properly, may address some of the realistic barriers, such as the identified low use of electronic vaccination records due to inconvenience [51]. On the other hand, we are not the only group that identifies the importance of making health information movable, reusable, and interoperable. Balog laid out the operational steps needed to exchange vaccination information between healthcare providers and public health authorities in 2012 [53]. The CDC IZ Gateway [28] might benefit from Balog’s publication, given that the paper was published in 2012 and the IZ Gateway went live in 2019. Other vaccine records-related examples include Canada and Asia [54,55,56]; however, they were all at the preparation or feasibility stages, and none have realized interoperable vaccine records.

## 4. Current Challenges in Achieving Interoperable Vaccination Records

The technical challenge is the first, given the inherent complexity of implementation and medicine. For those simple, straightforward scenarios, the current technology and infrastructure are adequate to achieve interoperable vaccination records. However, for more complicated cases, for example, for someone who has a compromised immune system or someone who is susceptible to allergies, then their vaccination records can become more similar to a comprehensive electronic health record, which is much more challenging to build in a computable manner, i.e., it *can be used by the receiver system and can be incorporated and consolidated into the records in the receiver system*. Furthermore, incomplete patient records can pose an additional challenge to determining whether a vaccine should be administered. An incomplete patient history was identified as a primary challenge three decades ago by Perry Miller and his colleagues [41]. Unfortunately, we still face the same or even worse challenges today due to many more operational information systems, many of which operate in silos.

Operational-level policy discrepancies [54] and the high priority of the confidentiality of individual health information pose additional challenges. This is particularly true in the USA since each state can pass its own legislation and establish its own policies on these issues. The lack of harmonization across states can pose operational challenges even at the most basic level, such as determining who can do what for what purposes with respect to interoperable vaccination records. This is a realistic context to navigate in order to achieve interoperable vaccination records. How to balance policymaking and legislation between individual states and the federal government regarding vaccination to produce harmonized, rational, and consistent legislation and policies for the nation is beyond the scope of this paper. Similar contexts between individual states and the federal government can be found between different countries. For example, among the Nordic countries, each country has its own legal regulations, which make interoperable vaccination records across national borders much more challenging. Therefore, we acknowledge that policy-level challenges exist at regional, national, and international levels, adding an additional layer of complexity at both the policy and operational levels. Given that more and more people travel and relocate in their lifetimes, this scenario is becoming increasingly common, and vaccination records serve as a form of health identity. However, without an interoperable vaccination record, this identity can fall through the cracks amid competing priorities.

The social and cultural challenges surrounding vaccination can serve as indirect barriers to interoperable vaccination records. This category of challenge includes individuals who are vaccination-hesitant [57,58] due to a lack of trust in institutions (healthcare professionals are a proxy for the institution), prior adverse events, prior severe allergic reactions, religious reasons, or other faith-based practices. Vaccination records will be unnecessary for individuals without vaccination, not to mention interoperable vaccination records. On the one hand, we want to respect individuals’ choices on this issue; on the other hand, we want to ensure they are well-informed about their decisions and the potential consequences. In addition, our scientific community has an obligation to better understand vaccine-induced allergic reactions by examining their mechanisms, their individual differences, and the reasons for these differences. Such insights can provide precise guidance on identifying the susceptible population who should take extra caution when receiving vaccination.

Political barriers, seemingly irrelevant on the surface, can be another challenge for interoperable vaccination records. Ideally, individual vaccination is a medical issue with consideration of social responsibility; group vaccination is a public health issue with consideration of individual circumstances. Unfortunately, too many people politicize the issue and weaponize vaccination, especially in the USA in recent years; furthermore, they put political labels on pro- and con-vaccine groups, completely regardless of the medical merits of vaccines and the critical role of vaccination in keeping individuals and communities free from preventable and contagious diseases, such as measles outbreaks in recent years [59,60]. If a significant percentage of the population chooses not to vaccinate for non-medical or non-religious reasons, i.e., below the needed percentage of vaccinated individuals to achieve herd immunity, then an interoperable vaccination record may not be the priority. Therefore, better educating the general public and exploring new ways to improve their health literacy should be given a higher priority to overcome this challenge.

Cultural political barriers seem much larger and more challenging than technical barriers. The latter can achieve predictable progress through diligence, resource allocation, process optimization, and consensus on the necessary infrastructure. The former, however, is much more challenging to make meaningful progress in a short time, particularly in this “generative information” era. The generative, unverified information can easily outweigh the authentic information regarding the volume and speed of the spread, e.g., misinformation can lead to vaccine hesitancy or even anti-vaccination. This reality presents new challenges for a topic related to everyone such as vaccination.

## 5. Moving Forward

**Figure 4:**
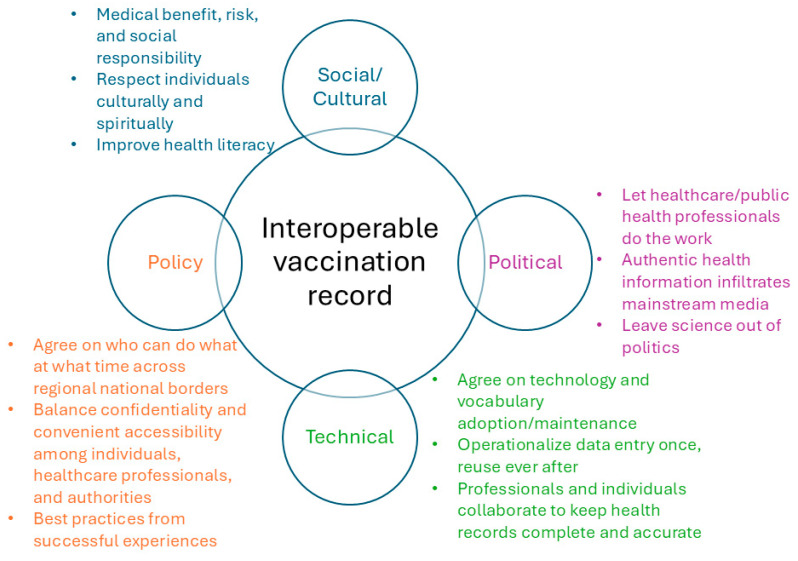
A roadmap to achieve interoperable vaccination records.

Researchers have proposed a simple and standard version for the vaccination record [61], and most patients may benefit from such a record. However, a small percentage of individuals require more comprehensive records to capture all the features relevant to their vaccination history, such as specific diagnoses or laboratory test results indicating a compromised immune system. Meanwhile, some successful experiences in practice, particularly from the Nordic countries, the United Kingdom, and Canada [54], can inform best practices moving forward. In summary, to achieve interoperable vaccination records, we need to make progress in the following aspects simultaneously: social and cultural, political, technical, and policy (Figure 4).

Regarding the social/cultural aspect, it is critical to balance medical benefit, risks, social responsibility, individual cultural choices, and spiritual practices. To achieve optimal outcomes, a practical solution is to improve health literacy among the general public, ensuring that every vaccination-related decision is well-informed and unbiased. Large-scale health education may require novel content and diverse delivery methods to reach a broader audience. Local vaccination rates can inform the design of a more precise health education campaign. A high vaccination coverage is necessary to achieve the required herd immunity for most infectious and preventable diseases; therefore, education and awareness campaigns about vaccination should focus on both the individual and community levels to amplify their effects.

Regarding the political aspect, the key points include at least the following: leave medicine, public health, and science out of the political battlefield; let healthcare professionals fulfill their roles without labeling them politically; and ensure mainstream media is full of authentic scientific information relevant to vaccination, i.e., minimize the generation of misinformation of vaccination and its distribution among the general public, especially in a systematic manner. This requires large social media companies to fulfill their social responsibility at the algorithmic level by combating misinformation, but not to rely entirely on voluntarism. The necessary legislation and any consequences of violations should be explicitly established to ensure optimal outcomes.

On the technical side, agreeing on the data standards and controlled vocabularies used to capture vaccine records is essential; though it may seem straightforward, it can be very time-consuming. Given the variety of data standards and controlled vocabularies and their ever-expanding scope, a more granular consensus is necessary, such as a minimal information model [62] for vaccine records, which could serve as a starting point. In addition, the stakeholders also need to agree on what constitutes a comprehensive vaccine record (including the fields, the values that can be used in the fields, and the metadata of the comprehensive records), where to save the external vaccine records, and what to do if there are discrepancies between the external and internal records or between two external records. Well-documented, accurate, and complete vaccination records are critical, but achieving them is not a simple task; operationalizing data reuse to achieve once-entry, multiple-reuse whenever needed is essential.

On the policy side, the work needs to take place at different levels: national, international, vendors, and institutional. The policies at the different levels must not only be in place but also be aligned with one another. In the USA, state-level legislation also plays a critical role in achieving interoperability. At a minimum, agreeing on who can do what and when across regional, national, and international borders is a prerequisite for interoperable vaccination records; moreover, balancing individual confidentiality, user convenience, and broad accessibility for citizens, healthcare providers, and authorities is necessary. From a more practical perspective, consensus among vendors and institutional-level policies can be a key facilitator or accelerator to achieve interoperable vaccine records. For example, agreeing on a minimal information model for vaccine records and on the standards (at the level of data elements) for the model’s metadata can be a concrete first step. Last but not least, citizens must agree to share their vaccination records; without their consent, Figure 3 will lose a third of the stakeholders. Meanwhile, the consent per se requires careful procedural and language preparation, such as when to provide the consent, how to provide it, and whether the vaccination information sharing is part of a broader health information sharing or requires a separate consent.

Figure 4 summarizes the key points for each aspect, and the challenge section provides further elaboration. Where there is a challenge, there are opportunities to overcome the challenge. Regarding an interoperable vaccination record, it is simpler than a complete interoperable electronic health record, but it is more complicated than reusable CDSS rules. More importantly, we are unlikely to achieve this through purely technical advances; rather, all the aspects shown in Figure 4 must progress in a coherent, interconnected manner to enable interoperable vaccination records for all.

## 6. Conclusions

Interoperable vaccine records are needed, not only in the USA, which is not one of the front-runners in interoperability, but also in the Nordic countries and the UK, both of which are advanced in keeping vaccination records and consolidating them for their citizens, respectively. Although achieving interoperable vaccine records is technically feasible and clinically important, the actual and large-scale implementation is not a simple and easy task in the midst of the social and political challenges, and it is premature to establish a day-by-day action plan before all the prerequisites are met.

## Data Availability

Please contact the corresponding author for any data-related inquiries.

## References

[1] WHO Organization WHO: Health Topics: Vaccines and Immunization. https://www.who.int/health-topics/vaccines-and-immunization#tab=tab_1.

[2] CDC Immunization Schedules. https://www.cdc.gov/vaccines/imz-schedules/index.html.

[3] Norwegian Institute of Public Health Norwegian Immunisation Registry SYSVAK. https://www.fhi.no/en/va/norwegian-immunisation-registry-sysvak/.

[4] Grove Krause T., Jakobsen S., Haarh M., Mølbak K. (2012). The Danish vaccination register. Euro Surveill..

[5] TRIFORK The Danish Vaccination Registry. https://trifork.com/?cases=ddv.

[6] Iceland Directorate of Health Vaccination Registration. https://island.is/en/vaccination-overview/skraning-bolusetningar.

[7] Baum U., Sundman J., Jääskeläinen S., Nohynek H., Puumalainen T., Jokinen J. (2017). Establishing and maintaining the National Vaccination Register in Finland. Euro Surveill..

[8] European Commission eHealth Swedish National Vaccination Register (Svevac). https://interoperable-europe.ec.europa.eu/collection/ehealth/document/swedish-national-vaccination-register-svevac.

[9] UK NHS View Your GP Health Record. https://www.nhs.uk/nhs-services/gps/view-your-gp-health-record/.

[10] Payne T.H., Detmer D.E., Wyatt J.C., Buchan I.E. (2011). National-scale clinical information exchange in the United Kingdom: Lessons for the United States. J. Am. Med. Inf. Assoc..

[11] UK NHS Digital Medicines Interoperability. https://digital.nhs.uk/services/digital-and-interoperable-medicines.

[12] Shortliffe E.H., Cimino J.J., Chiang M.F. (2021). Biomedical Informatics: Computer Applications in Health Care and Biomedicine.

[13] Hersh W. (2022). Health Informatics: Practical Guide.

[14] Everson J., Adler-Milstein J., Phillips R.L., Bazemore A.W., Patel V. (2025). EHR Interoperability Experiences Reported by Family Physicians. JAMA Netw. Open.

[15] Virkkunen S., Granlund T., Kaikkonen R. (2025). Implementing a Cost-Efficient and Interoperable Health Data Infrastructure: A Multi-Region Finnish Case Study. Stud. Health Technol. Inf..

[16] Mullie T., Chuck A., Razak F. (2025). A Health Economic Analysis of the Potential in Transforming Canada’s Health Data Systems. Healthc. Q..

[17] Li E., Lounsbury O., Hasnain M., Ashrafian H., Darzi A., Neves A.L., Clarke J. (2025). Physician experiences of electronic health record interoperability and its practical impact on care delivery in the English NHS: A cross-sectional survey study. BMJ Open.

[18] Dombkowski K.J., Patel P.N., Peng H.K., Cowan A.E. (2025). The Effect of Electronic Health Record and Immunization Information System Interoperability on Medical Practice Vaccination Workflow. Appl. Clin. Inf..

[19] Bastola N.D., Tcheng J.E., Schlossman D.M., Windle J.R. (2025). Framework for Improving Patient Safety: Reference Model for FHIR-Enabled, Patient-Centric Home Medication List Management and Medication Reconciliation. Appl. Clin. Inf..

[20] Adams K.T., Howe J.L., Fong A., Puthumana J.S., Kellogg K.M., Gaunt M., Ratwani R.M. (2017). An Analysis of Patient Safety Incident Reports Associated with Electronic Health Record Interoperability. Appl. Clin. Inf..

[21] Harrington L. (2018). Interoperability of Infusion Pumps with Electronic Health Records. AACN Adv. Crit. Care.

[22] Goldman J.M., Weininger S., Jaffe M.B. (2020). Applying Medical Device Informatics to Enable Safe and Secure Interoperable Systems: Medical Device Interface Data Sheets. Anesth. Analg..

[23] Andrew Fang H.S. (2021). Commercially Successful Blockchain Healthcare Projects: A Scoping Review. Blockchain Healthc. Today.

[24] CDC: Immunization Information Systems (IIS) Immunization Information Systems Resources. https://www.cdc.gov/iis/about/index.html.

[25] CDC: Immunization Information Systems (IIS) Contacts for IIS Immunization Records. https://www.cdc.gov/iis/contacts-locate-records/index.html.

[26] South Carolina Department of Public Health SIMON: Statewide Immunization Online Network. https://simon.dhec.sc.gov/simon/Login.aspx.

[27] Indiana Department of Health CHIRP: Children and Hoosier Immunization Registry Program. https://chirp.in.gov/.

[28] CDC: Immunization Information Systems (IIS) Immunization (IZ) Gateway. https://www.cdc.gov/iis/iz-gateway/index.html.

[29] Office of the National Coordinator for Health Information Technology Adoption of Electronic Health Records by Hospital Service Type 2019–2021. https://www.healthit.gov/data/quickstats/adoption-electronic-health-records-hospital-service-type-2019-2021.

[30] CDC: National Center for Health Statistics National Electronic Health Records Survey. https://www.cdc.gov/nchs/nehrs/results/index.html.

[31] HealthIT.gov Health IT Quick Stats. https://www.healthit.gov/data/quickstats.

[32] Health Level Seven International (HL7). About HL7. https://www.hl7.org/about/.

[33] FHIR H. HL7 FHIR: Release 5. https://www.hl7.org/fhir/overview.html.

[34] SNOMED International SNOMED. https://www.snomed.org/.

[35] Regenstrief Institute LOINC from Regenstrief. https://loinc.org/.

[36] Miller P.L., Frawley S.J., Sayward F.G., Yasnoff W.A., Duncan L., Fleming D.W. (1996). IMM/Serve: A rule-based program for childhood immunization. Proc. AMIA Annu. Fall Symp..

[37] Brandt C.A., Frawley S.J., Powsner S.M., Shiffman R.N., Miller P.L. (1997). Visualizing the logic of a clinical guideline: A case study in childhood immunization. Methods Inf. Med..

[38] Miller P.L., Frawley S.J., Sayward F.G., Yasnoff W.A., Duncan L., Fleming D.W. (1997). Combining tabular, rule-based, and procedural knowledge in computer-based guidelines for childhood immunization. Comput. Biomed. Res..

[39] Miller P.L. (1998). Tools for immunization guideline knowledge maintenance. I. Automated generation of the logic “kernel” for immunization forecasting. Comput. Biomed. Res..

[40] Miller P.L., Frawley S.J., Sayward F.G. (1998). Issues in accommodating national changes and local variation in a computer-based guideline for childhood immunization and in related knowledge maintenance tools. Proc. AMIA Symp..

[41] Miller P.L., Frawle S., Sayward F.G. (1999). Exploring three approaches for handling incomplete patient histories in a computer-based guideline for childhood immunization. Proc. AMIA Symp..

[42] Miller P.L. (2001). Domain-constrained generation of clinical condition sets to help test computer-based clinical guidelines. J. Am. Med. Inf. Assoc..

[43] Miller P.L., Frawley S.J., Sayward F.G. (2001). Maintaining and incrementally revalidating a computer-based clinical guideline: A case study. J. Biomed. Inf..

[44] Arzt N.H., Chertcoff D., Nicolary S., Suralik M., Berry M. (2021). Immunization calculation engine: An open source immunization evaluation and forecasting system. Learn. Health Syst..

[45] Wilson K., Atkinson K.M., Penney G. (2015). Development and release of a national immunization app for Canada (ImmunizeCA). Vaccine.

[46] Orlioglu S., Boobalan A.S., Abanyie K., Boyce R.D., Min H., Gong Y., Sittig D.F., Biondich P., Wright A., Nøhr C. (2025). Reusable Generic Clinical Decision Support System Module for Immunization Recommendations in Resource-Constraint Settings. AMIA Summit 2025.

[47] Jing X., Min H., Gong Y., Ernst M.A., Weaver A., Crozier C., Robinson D., Sittig D.F., Biondich P.G., Orlioglu S. (2025). Vaccination Schedules Recommended by the Centers for Disease Control and Prevention: From Human-Readable to Machine-Processable. Vaccines.

[48] Jing X., Goli R., Komatineni K., Alluri S., Hubig N., Min H., Gong Y., Sittig D.F., Biondich P., Robinson D. (2024). Active Learning Pipeline to Identify Candidate Terms for a CDSS Ontology. Stud. Health Technol. Inf..

[49] Goli R., Hubig N., Min H., Gong Y., Sittig D.F., Robinson D., Biondich P., Wright A., Nøhr C., Law T. (2023). Keyphrase Identification Using Minimal Labeled Data with Hierarchical Context and Transfer Learning. MedRxiv.

[50] Jing X., Min H., Gong Y., Cimino J., Robinson D., Sittig D., Biondich P., Wright A., Nøhr C., Law T. (2021). A clinical decision support system (CDSS) ontology to facilitate portable vaccination CDSS rules: Preliminary results. AMIA 2021.

[51] Sorg K., Khobzi H. (2022). A decade of the Swiss electronic vaccination Record: Some insights based on an exploratory data analysis. Int. J. Med. Inf..

[52] Wilson K., Atkinson K.M., Bell C.P. (2016). Travel Vaccines Enter the Digital Age: Creating a Virtual Immunization Record. Am. J. Trop. Med. Hyg..

[53] Balog J. (2012). A Three-Step Approach for Creating Successful Electronic Immunization Record Exchanges between Clinical Practice and Public Health. Online J. Public Health Inf..

[54] Rubens-Augustson T., Wilson L.A., Bell C., Wilson K. (2025). Building the foundation for immunization information system interoperability: Lessons from the Canadian context. Health Inform. J..

[55] Duszynski K.M., Stark J.H., Cohet C., Huang W.T., Shin J.Y., Lai E.C., Man K.K.C., Choi N.K., Khromava A., Kimura T. (2021). Suitability of databases in the Asia-Pacific for collaborative monitoring of vaccine safety. Pharmacoepidemiol. Drug Saf..

[56] Heryawan L., Mori Y., Yamamoto G., Kume N., Lazuardi L., Fuad A., Kuroda T. (2025). Fast Healthcare Interoperability Resources (FHIR)-Based Interoperability Design in Indonesia: Content Analysis of Developer Hub’s Social Networking Service. JMIR Form. Res..

[57] Lafnitzegger A., Gaviria-Agudelo C. (2022). Vaccine Hesitancy in Pediatrics. Adv. Pediatr..

[58] Cataldi J.R., O’Leary S.T. (2021). Parental vaccine hesitancy: Scope, causes, and potential responses. Curr. Opin. Infect. Dis..

[59] Durrheim D.N. (2024). Never waste a measles outbreak. Commun. Dis. Intell..

[60] Bagcchi S. (2024). A measles outbreak in a school. Lancet Infect. Dis..

[61] Maurer W., Seeber L., Rundblad G., Kochhar S., Trusko B., Kisler B., Kush R., Rath B. (2014). Vienna Vaccine Safety Initiative, Standardization and simplification of vaccination records. Expert. Rev. Vaccines.

[62] Hochheiser H., Jing X., Garcia E.A., Ayvaz S., Sahay R., Dumontier M., Banda J.M., Beyan O., Brochhausen M., Draper E. (2021). A Minimal Information Model for Potential Drug-Drug Interactions. Front. Pharmacol..

